# Chiral Helimagnetism and One‐Dimensional Magnetic Solitons in a Cr‐Intercalated Transition Metal Dichalcogenide

**DOI:** 10.1002/adma.202101131

**Published:** 2021-07-24

**Authors:** Chenhui Zhang, Junwei Zhang, Chen Liu, Senfu Zhang, Ye Yuan, Peng Li, Yan Wen, Ze Jiang, Bojian Zhou, Yongjiu Lei, Dongxing Zheng, Chengkun Song, Zhipeng Hou, Wenbo Mi, Udo Schwingenschlögl, Aurélien Manchon, Zi Qiang Qiu, Husam N. Alshareef, Yong Peng, Xi‐Xiang Zhang

**Affiliations:** ^1^ Physical Science and Engineering Division (PSE) King Abdullah University of Science and Technology (KAUST) Thuwal 23955‐6900 Saudi Arabia; ^2^ Key Laboratory for Magnetism and Magnetic Materials of Ministry of Education School of Physical Science and Technology and Electron Microscopy Centre of Lanzhou University Lanzhou University Lanzhou Gansu Province 730000 China; ^3^ Songshan Lake Materials Laboratory Dongguan Guangdong 523808 China; ^4^ Guangdong Provincial Key Laboratory of Optical Information Materials and Technology & Institute for Advanced Materials South China Academy of Advanced Optoelectronics South China Normal University Guangzhou Guangdong Province 510006 China; ^5^ National Center for International Research on Green Optoelectronics South China Normal University Guangzhou Guangdong Province 510006 China; ^6^ Tianjin Key Laboratory of Low Dimensional Materials Physics and Processing Technology Institute of Advanced Materials Physics Faculty of Science Tianjin University Tianjin Tianjin Municipality 300354 China; ^7^ Aix‐Marseille Université CNRS CINaM Marseille 13288 France; ^8^ Department of Physics University of California Berkeley CA 94720 USA

**Keywords:** chiral soliton lattice, Dzyaloshinskii–Moriya interaction, magnetic solitons, topological spin textures, two‐dimensional materials

## Abstract

Chiral magnets endowed with topological spin textures are expected to have promising applications in next‐generation magnetic memories. In contrast to the well‐studied 2D or 3D magnetic skyrmions, the authors report the discovery of 1D nontrivial magnetic solitons in a transition metal dichalcogenide 2*H*‐TaS_2_ via precise intercalation of Cr elements. In the synthetic Cr_1/3_TaS_2_ (CTS) single crystal, the coupling of the strong spin–orbit interaction from TaS_2_ and the chiral arrangement of the magnetic Cr ions evoke a robust Dzyaloshinskii–Moriya interaction. A magnetic helix having a short spatial period of **≈**25 nm is observed in CTS via Lorentz transmission electron microscopy. In a magnetic field perpendicular to the helical axis, the helical spin structure transforms into a chiral soliton lattice (CSL) with the spin structure evolution being consistent with the chiral sine‐Gordon theory, which opens promising perspectives for the application of CSL to fast‐speed nonvolatile magnetic memories. This work introduces a new paradigm to soliton physics and provides an effective strategy for seeking novel 2D magnets.

## Graphical abstract

Chiral helimagnetism and 1D magnetic solitons are discovered in Cr‐intercalated 2*H*‐TaS_2_. The chiral helimagnetic structure has an initial period of ≈25 nm, and it transforms into a chiral soliton lattice in an external magnetic field. The spatial period evolution of this topological spin texture obeys the chiral sine‐Gordon theory, revealing great potential for fast‐speed racetrack memories.

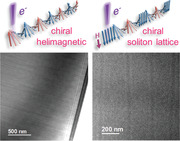

## 1 Introduction

Chiral spin textures are typically discovered in noncentrosymmetric systems and are stabilized due to the competition between exchange interaction, magnetic anisotropy, and Dzyaloshinskii–Moriya (DM) interaction.^[^ ^1^, ^2^ ^]^ They are expected to be promising candidates for the fabrication of novel magnetic memories that integrate high storage density, fast processing speed, and low energy consumption.^[^ ^3^, ^4^ ^]^ Van der Waals (vdW) materials have stable crystal structures even down to atomic thickness and show great compatibility for assembling artificial heterostructures,^[^ ^5^ ^]^ rendering them as ideal platforms for developing such memories. A central question is how to induce chiral magnetism in vdW structures. Although the recently discovered vdW ferromagnets, such as Cr_2_Ge_2_Te_6_,^[^ ^6^ ^]^ CrI_3_,^[^ ^7^ ^]^ and Fe*_x_* GeTe_2_ (*x* = 3, 4),^[^ ^8^, ^9^, ^10^, ^11^ ^]^ possess intrinsic long‐range magnetic order, they belong to centrosymmetric space groups; thus, crystal chirality is absent in these materials. An alternative way to induce magnetism is to intercalate magnetic elements within the vdW gaps of non‐magnetic vdW materials, such as transition metal dichalcogenides (TMDCs).^[^ ^12^ ^]^ In this manner, the intercalators located at the ordered positions weakly bond with the adjacent chalcogenide slabs,^[^ ^13^ ^]^ which may lead to magnetic coupling through the Ruderman–Kittel–Kasuya–Yosida interaction among the magnetic ions.^[^ ^14^ ^]^ Similar to conventional vdW magnets, the intercalated TMDCs are cleavable and maintain long‐range magnetic order even down to 2D scale.^[^ ^15^, ^16^, ^17^ ^]^ More importantly, if the intercalator concentration is precisely controlled at *x* = 1/3 in T*_x_* MX_2_ (T = V, Cr, Mn, Fe, Co, Ni; M = Nb, Ta; X = S, Se), the entire system forms a noncentrosymmetric chiral crystal structure (*P*6_3_22 space group), although its parent compound MX_2_ is centrosymmetric non‐chiral.^[^ ^18^ ^]^ The amalgamation of long‐rang magnetic order and crystal chirality in T*_x_* MX_2_ may promote interesting spin textures, such as magnetic solitons.^[^ ^19^, ^20^ ^]^

**Figure 1:**
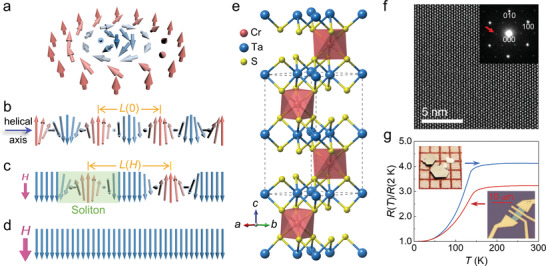
Different types of spin textures and basic physical properties of CTS. a) Schematic diagram of a chiral skyrmion (Bloch‐type) that exists in certain B20 magnets. b) A CHM spin structure propagating along its helical axis. *L*(0) denotes the zero‐field spatial period of the CHM spin structure. c) The CSL state formed in an external magnetic field applied perpendicular to the helical axis. *L*(*H*) denotes the field‐dependent spatial period of CSL. A soliton is highlighted by a green background. d) The FFM state formed in a magnetic field larger than *H*_s_. e) Schematic diagram of the CTS crystal structure, where the dashed lines indicate the unit cell. f) HRTEM and electron diffraction patterns taken along [001] orientation. The red arrow points at a (1/3 1/3 0)‐type reciprocal spot. g) Temperature‐dependent resistance of CTS bulk (blue) and nanosheet (red) samples. The grid size in the bulk sample photo is 1 mm^2^.

A magnetic soliton is a 1D topologically nontrivial spin texture.^[^ ^20^, ^21^ ^]^ Similar to the well‐known magnetic skyrmions,^[^ ^22^, ^23^, ^24^ ^]^ magnetic solitons also derive from the chiral helimagnetic (CHM) ground state induced by the DM interaction. Schematic diagrams of a 2D Bloch‐type skyrmion and 1D CHM spin structure are shown in **Figure** **1**a,b, respectively. However, the evolutions of solitons and skyrmions under an external magnetic field vary from one another. When the external magnetic field is perpendicular to the helical axis, ferromagnetic domains are generated with the spins inside the domains being aligned to the field direction, and more importantly, they are separated by a series of 2π magnetic twists, that is, the so‐called magnetic solitons^[^ ^20^ ^]^ (Figure 1c). The chiral soliton lattice (CSL) is defined as the superlattice of solitons and ferromagnetic domains.^[^ ^20^ ^]^ As a particle‐like excitation, the dynamic control of solitons, including creation, annihilation, and motion, are expected to be realized via the application of a spin‐polarized current.^[^ ^25^, ^26^ ^]^ More importantly, by comparing the Klirr factors (the ratio of the third‐ to first‐harmonic ac magnetic response) of various non‐collinear spin textures, a recent study pointed out that the CSL exhibits stronger robustness of the spin texture against the external magnetic field than the skyrmion lattice.^[^ ^27^ ^]^ This provides a reasonable explanation for the fact that the CSL can survive in very wide temperature and field ranges in the phase diagram,^[^ ^28^, ^29^, ^30^ ^]^ which is essential for practical applications. Another important parameter for the fabrication of practical devices is the spatial size of the spin textures. Cr_1/3_NbS_2_ (CNS) is a paradigmatic chiral soliton crystal that has been verified via real‐space observation,^[^ ^20^, ^31^, ^32^ ^]^ and its helical period is ≈48 nm, which is comparable to the size of skyrmions but significantly smaller than that of micrometer‐sized trivial magnetic bubbles.^[^ ^24^, ^33^ ^]^ Further, helimagnetism was recently observed in vdW ferromagnet Fe_5_GeTe_2_, which is caused by the asymmetric arrangement of Fe–Ge pairs at split sites.^[^ ^34^ ^]^ Certain reports have claimed the observation of helical magnetic structures and solitons in Mn_1/3_NbS_2_ with the magnetic helix having a long pitch period of ≈250 nm.^[^ ^35^, ^36^ ^]^ A smaller spatial period could lead to a smaller device size and higher storage density in future memory devices.

The helical period is known to be proportional to the ratio of exchange interaction to DM interaction.^[^ ^37^ ^]^ Considering that the DM interaction originates from relativistic spin–orbit coupling (SOC),^[^ ^2^ ^]^ one conceivable way to reduce the helical period is to strengthen the SOC of the chiral system. In this study, we chose TaS_2_ as the parent material to construct a new chiral magnet because TaS_2_ has a larger atomic SOC contribution from the heavy Ta atoms in comparison with other MX_2_ candidates.^[^ ^38^ ^]^ The Cr atoms were successfully intercalated into the vdW gaps of TaS_2_ via the vapor transport method, and the obtained magnetic Cr_1/3_TaS_2_ (CTS) crystal possessed a noncentrosymmetric chiral crystal structure. The anomalous temperature‐ and field‐dependent magnetization behavior provides indirect evidence for the helimagnetic nature of CTS. Using Lorentz transmission electron microscopy (L‐TEM), we achieved the direct observation of nontrivial spin textures in CTS and found that they were topologically protected against the perturbation of extrinsic defects. Moreover, we established a comprehensive phase diagram based on the results of the bulk magnetization measurements and L‐TEM images. CTS has a short helical period of ≈25 nm and a very wide soliton phase region. Endowed with the exotic features, CTS is expected to be a new paradigm for the soliton physics and low‐dimensional spintronic applications.

## 2 Results and Discussion

### 2.1 Crystal Structure and Transport Properties

The chemical composition of the CTS single crystal was characterized by energy dispersive X‐ray spectroscopy (EDS; Figure S1, Supporting Information), which was determined to be Cr_0.33_TaS_2.06_. Its crystal structure illustrated in Figure 1e shows that the Cr atoms occupy the octahedral sites between two 2*H*‐TaS_2_ layers, and the entire crystal forms a layered structure in a sequence of Ta‐S‐Cr‐S‐Ta‐… slabs along the *c* direction. A high‐resolution transmission electron microscopy (HRTEM) image obtained along the [001] orientation is presented in Figure 1f. Previous research has shown that, in 3*d* transition‐metal intercalated TMDCs, the magnetic atoms tend to form 2 × 2 and $\sqrt{3}\times \sqrt{3}$ ordered superlattices with concentrations of 1/4 and 1/3, respectively, corresponding to the centrosymmetric *P*6_3_/*mmc* and noncentrosymmetric chiral *P*6_3_22 space groups.^[^ ^18^ ^]^ In the corresponding electron diffraction pattern, (1/3 1/3 0)‐type reciprocal spots were observed, confirming the scenario of $\sqrt{3}\times \sqrt{3}$ superlattice.^[^ ^18^ ^]^ Hence, the confirmed crystal chirality and strong SOC from TaS_2_ are expected to evoke a considerable DM interaction in CTS. The axis of the magnetic helicity is expected to be along the *c* axis of the crystal with the spins in the *ab* plane. Bulk CTS exhibits metallic behavior over the measured temperature range of 2–300 K, as shown in Figure 1g. The temperature‐dependent resistance reduces rapidly below ≈140 K. Because this temperature is approximately similar to the magnetic phase transition temperature (*T*_c_ discussed in the next section) of CTS, the sharp decrease in resistance at ≈140 K probably correlates with the onset of spin ordering, which suppresses electron scattering. Similar to conventional vdW materials, bulk CTS crystals can be thinned down to 2D nanosheets via mechanical exfoliation. The temperature‐dependent resistance of a 28‐nm‐thick sample is also exhibited in Figure 1g for comparison (Figure S3, Supporting Information, for the atomic force microscope image). It is evident that the 28‐nm‐thick sample shows nearly identical behavior as its bulk counterpart, in particular, the sharp decrease at ≈140 K. This may imply that the magnetic ordering of CTS is well‐preserved till the bulk transition temperature in the 2D nanosheet.

### 2.2 Evidence of Chiral Soliton Lattice Exhibited in Magnetic Properties

**Figure 2:**
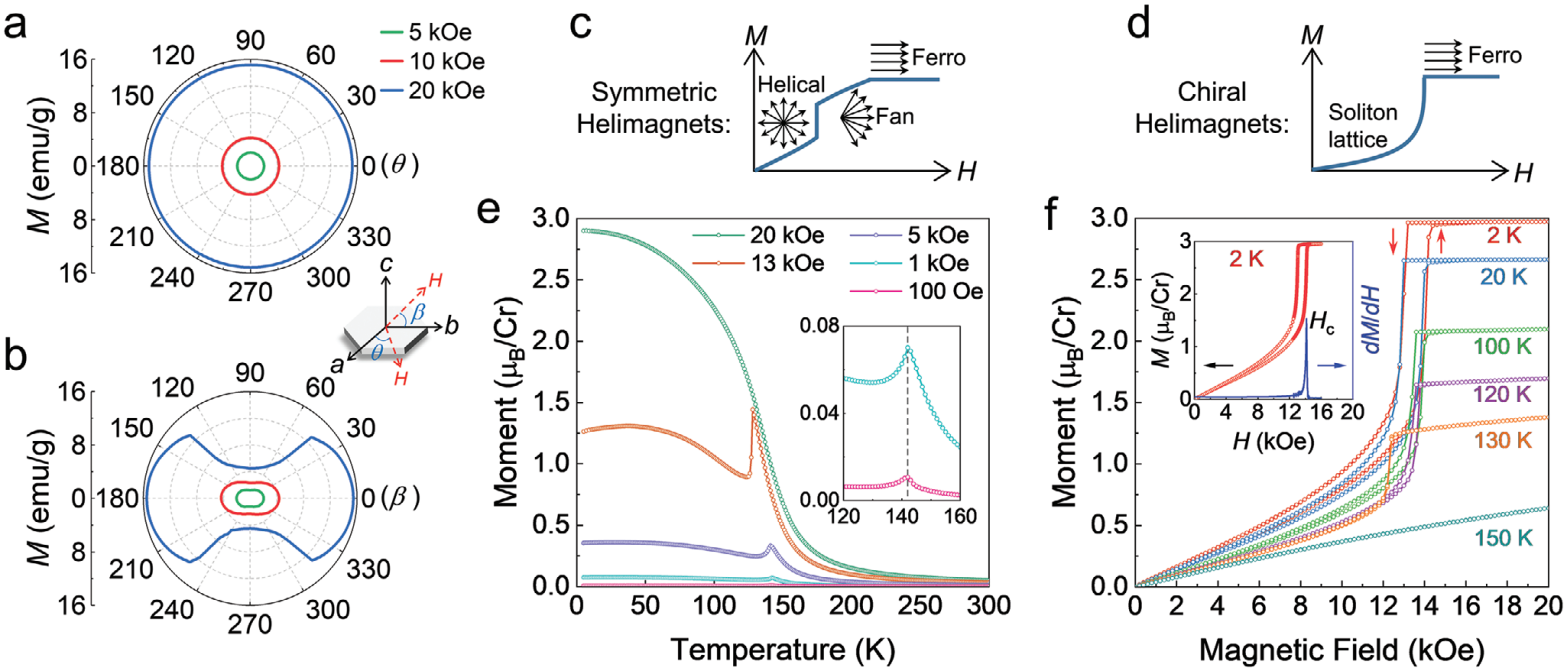
Magnetization results of a CTS bulk single crystal. a,b) Angular‐dependent magnetizations measured in the *ab* (a) and *bc* (b) planes at 100 K with magnetic fields of 5, 10, and 20 kOe. c,d) Schematic diagrams of the initial isothermal magnetization curves of Yoshimori‐type (c) and Dzyaloshinskii‐type (d) helimagnets. The magnetic field is applied perpendicular to the helical axis. e) Temperature‐dependent magnetization of CTS measured with zero‐field‐cooled protocol at various magnetic fields applied in the *ab* plane. The inset shows the magnified low‐field *M*(*T*) curves where the vertical dashed line indicates 142 K. f) *ab*‐plane magnetization measured at various temperatures by sweeping the field between 0 and 20 kOe. The inset shows the 2 K *M*(*H*) curve measured with small field steps (red) and its differential magnetization for the increasing field (blue). The peak position in the d*M*/d*H* curve is defined as *H*_c_.

First, we examined the magnetic anisotropy of the CTS bulk single crystal. The angular‐dependent magnetizations measured at 100 K under magnetic fields of 5, 10, and 20 kOe are shown in **Figure** **2**a,b, where θ and β are the *ab*‐ and *bc*‐plane rotation angles, respectively. The curves show a twofold symmetry in the *bc* plane, and the magnetization is significantly suppressed when the magnetic field passes through the *c* axis, while the anisotropy in the *ab* plane is negligibly small. In addition, we measured the magnetization of CTS by sweeping the field between −70 and 70 kOe at various temperatures (Figure S4, Supporting Information), wherein no coercive field was detected for both *H*//*ab* and *H*//*c* configurations. The *ab*‐plane magnetization was quickly saturated at 2 K with saturation moment being 2.97 *μ_B_*, corresponding to the trivalent state of Cr^3+^. However, at the same temperature, the moment was not saturated even under a field up to 70 kOe when *H*//*c*. Thus, the CTS single crystal has a magnetic easy plane (the *ab* plane) and a hard axis along the *c* direction.

Figure 2e shows the temperature dependence of the magnetization measured with the zero‐field‐cooled protocol at various magnetic fields applied in the *ab* plane. When the magnetic field is not sufficiently strong (below 13 kOe), a characteristic peak can be observed in the *M*(*T*) curves. Similarly, this intriguing feature has been observed in certain skyrmion crystals, where the peak is considered a signature of the onset of the skyrmion phase.^[^ ^39^, ^40^ ^]^ We also notice that the peak moves toward higher temperatures as the applied field decreases (Figure 2e and Figure S5, Supporting Information). However, it stays at 142 K for all curves obtained with a magnetic field lower than 1 kOe. Thus, 142 K is considered the transition temperature of CTS.^[^ ^21^, ^41^ ^]^ Alternatively, the first derivative of the 50 Oe field‐cooled *M*(*T*) curve (d*M*/d*T*) indicates a *T*_c_ of 144 K (Figure S6, Supporting Information), which is very close to 142 K, the peak temperature in the low‐field *M*(*T*) curves. Therefore, the magnetic phase transition temperature should be in the range of 142–144 K, which is consistent with the resistivity result shown in Figure 1g. Figure 2f displays the *ab*‐plane magnetization measured at various temperatures by sweeping the field between 0 and 20 kOe. Below *T*_c_, the *M*(*H*) curves exhibit a downward convex shape with an anomaly at a certain critical field *H*_c_, where the magnetization abruptly increases with further increase in *H*. Such abrupt increase in magnetization was initially observed in CNS and was understood as a first‐order transition between helical and forced ferromagnetic states.^[^ ^37^ ^]^ Then a similar situation was found in Ba_2_CuGe_2_O_7_, where the existence of soliton lattice was demonstrated, and the sharp change in magnetization was interpreted as an incommensurate–commensurate phase transition.^[^ ^42^ ^]^ Moreover, at low temperatures, the field‐increasing and field‐decreasing curves do not overlap, indicating hysteretic behavior. The hysteresis loops below *T*_c_ show a characteristic sheared shape (also see Figure S4a, Supporting Information), and similar *M*(*H*) curves are often observed in skyrmion systems and are regarded as a typical signature of labyrinth domain states.^[^ ^43^, ^44^, ^45^ ^]^ Similarly, it is reasonable to expect that there exists an intermediate state before CTS becomes saturated and that a larger magnetic field is required to eliminate this state in the field‐increasing process. To understand the transition more clearly, we measured the *M*(*H*) curve at 2 K with much smaller field steps, as shown in the inset of Figure 2f. The corresponding d*M*/d*H* curve (blue) for the increasing field was also plotted to determine *H*_c_. The critical field *H*_c_ was ≈14.1 kOe with hysteresis width of ≈1.1 kOe at 2 K. As the temperature increased, both the critical field and hysteresis width decreased, which finally resulted in a linear magnetization curve when the temperature was above *T*_c_.

It should be noted that, when an external field is applied perpendicular to the helical axis, there is a significant difference between the spin structures of the Yoshimori‐type^[^ ^46^ ^]^ (symmetric non‐chiral) and Dzyaloshinskii‐type^[^ ^47^ ^]^ (chiral) helimagnets. In the former case, the symmetric helimagnetic structure experiences a discontinuous transition into a fan structure and finally proceeds smoothly into the forced ferromagnetic (FFM) state,^[^ ^37^ ^]^ as illustrated in Figure 2c. In contrast, in the latter case, such a fan structure is not expected to occur because of topological chirality, and it is substituted by the CSL state as shown in Figure 2d.^[^ ^48^, ^49^ ^]^ Obviously, the results in Figure 2f are consistent with the description of the Dzyaloshinskii‐type helimagnetic model. In the *H*//*c* configuration, the downward convex *M*(*H*) was not observed (Figure S4b, Supporting Information). Thus, the *M*(*H*) behavior indirectly confirms the existence of CHM spin structures in CTS, having the crystal *c* axis as its helical axis. Thus, the intermediate state mentioned above should be the CSL structure as described in the Dzyaloshinskii model.

### 2.3 Direct Observation of Chiral Spin Textures by Lorentz Transmission Electron Microscopy

**Figure 3:**
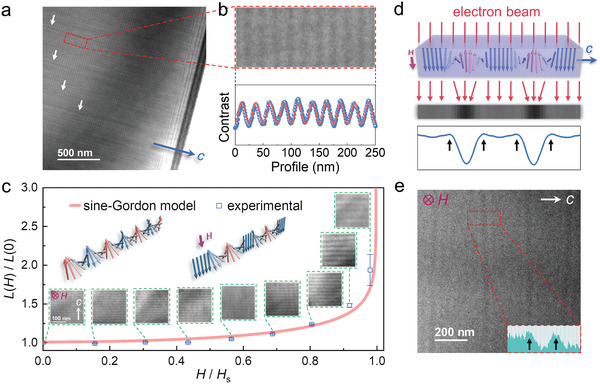
L‐TEM observations of chiral spin textures in CTS lamellar samples. a) A typical micrograph obtained at 94 K with zero magnetic field, where the white arrows indicate the scratch defects induced by the FIB. b) A magnified image captured from (a) as well as its contrast profile, where the blue dots and pink line are the observed contrast and fitted sinusoidal function, respectively. c) Magnetic‐field‐dependent spatial period of a CSL at 94 K. d) The simulated L‐TEM pattern of a left‐handed CSL in Fresnel under‐focused mode as well as its contrast profile. e) An L‐TEM image acquired at 92 K in a magnetic field of ≈15.4 kOe. The inset is the contrast profile in the selected area, wherein the black and white arrows indicate the contrast valley and peaks, respectively.

Thereafter, we employed L‐TEM, which has been demonstrated to be very successful in real‐space imaging of nanoscale spin textures,^[^ ^20^, ^24^, ^50^ ^]^ to acquire direct evidence of chiral magnetic structures in CTS. The lamellar samples were cut from bulk crystals along the *c* direction and thinned using the focused ion beam (FIB) milling technique (described in the Experimental Section). As expected, stripe patterns with alternating bright and dark contrast were observed in the images taken with the Fresnel under‐focused mode without an external magnetic field at 94 K, as shown in **Figure** **3**a. The stripes spread approximately all over the sample plane and remained strictly perpendicular to the *c* axis, but disappeared when the defocus value was reduced to zero. Figure 3b shows a magnified image of the magnetic pattern and its contrast profile. The contrast intensity is well‐fitted by the sinusoidal function (lower panel of Figure 3b), and a modulation period of ≈25 nm can be explicitly extracted. More interestingly, the sinusoidal modulation is quite robust, irrespective of the scratches induced by the FIB, as highlighted by the white arrows in Figure 3a, which strongly indicates that the observed magnetic texture is robust against the perturbation of extrinsic defects. Subsequently, we gradually increased the vertical magnetic field and examined the evolution of the spin structures. As shown in Figure 3c, the period of contrast increases monotonously with increasing magnetic field, and consequently the contrast pattern vanishes when *H* reaches the saturation field *H*_s_ (≈12.7 kOe), indicating that the system entered into the FFM state. It is known that ferromagnetic domains do not lead to L‐TEM contrast because there is no magnetization gradient inside the domains. The contrast pattern in the high‐field region shown in Figure 3e can be well‐interpreted by the formation of the CSL state: the wide gray stripes correspond to the ferromagnetic domains, while the narrow dark stripes correspond to the left‐handed magnetic solitons.^[^ ^20^ ^]^ The CSL spin structures can be obtained by micromagnetic simulations (details in the Experimental Section), and the illustrations of the CSLs with left‐ and right‐handed chiralities as well as their simulated L‐TEM contrasts are exhibited in Figure S7, Supporting Information. When an electron beam passes through the sample vertically, the electrons in the soliton region drift because of the Lorentz force. The left‐handed soliton converges the electrons, forming a bright zone on the Fresnel over‐focused micrograph. While, the ferromagnetic domain does not deflect electrons, forming a gray zone. The final contrast is reversed in the under‐focused mode, that is, the bright zone changes to a dark zone, as shown in Figure 3d. Furthermore, there should be two satellite contrast peaks laying astride in the dark zone in the under‐focused pattern, as indicated by the black arrows in the simulated contrast profile in Figure 3d. However, in practice, they are not easily resolved solely by the eyes; thus the contrast profile was extracted across the dark zone and has been presented in the inset of Figure 3e. As expected, the satellite contrast peaks can be recognized and indicated by the black arrows, confirming the emergence of magnetic solitons in CTS. In the CSL state, the Zeeman energy joins in the competition between the Heisenberg exchange that tends to form ferromagnetic domains and the DM interaction that favors the chiral helices. This competition ends when the Zeeman energy becomes dominant at a sufficient external magnetic field, which leads to ferromagnetic saturation. The field‐dependent spatial period of the CSL can be described by the 1D chiral sine‐Gordon model, and the inverse soliton density *L*(*H*)/*L*(0) is given by^[^ ^48^ ^]^

(1) $$\begin{array}{c} L(H)/L(0)\;\;=\;\;\frac{4K(\kappa )E(\kappa )}{\pi^{2}} \end{array}$$

where κ is the elliptic modulus and *K*(κ) and *E*(κ) are the complete elliptic integrals of the first and second kinds, respectively. *L*(*H*)/*L*(0) can be determined solely by the ratio of the applied field *H* to *H*_s_ using the relation of κ^2^/*E*(κ)^2^ = *H*/*H*_s_, and it continuously increases from 1 to infinity as *H* increases from zero (κ = 0) to *H_s_* (κ = 1). The theoretical curve of *L*(*H*)/*L*(0) was plotted against the experimental data and is shown in Figure 3c. The experimental data can be well‐described by the sine‐Gordon theory, which further demonstrates that the spin structure that appears in CTS is indeed the CSL. As the sine‐Gordon equation is Lorentz invariant, the CSL should experience Lorentz contraction, meaning that its width is expected to shrink at high velocities.^[^ ^51^, ^52^ ^]^ This is similar to situations in antiferromagnetic and ferrimagnetic domain walls.^[^ ^52^, ^53^, ^54^, ^55^ ^]^ In addition, owing to its topological protection, the CSL is expected to be free of the Walker breakdown and standout as a suitable candidate for fast‐speed racetrack memories.^[^ ^56^, ^57^ ^]^

The helical period of a CHM structure is closely related to the DM and exchange interactions in magnetic materials. The monoaxial DM interaction in CTS can be expressed as ***D*** ⋅ ***S***_i_ × ***S***_j_, where ***S***_i_ and ***S***_j_ are the neighboring spins in adjacent layers. The sign (negative or positive) of DM vector ***D*** determines the chirality (left‐ or right‐handed) of the CHM structure. Without the external magnetic field, the spins in adjacent layers are misaligned with a small pitch angle of tan^−1^(*D*/*J* ^∥^), where *J* ^∥^ is the interlayer ferromagnetic exchange interaction.^[^ ^58^ ^]^ So the modulation wave number of the helix is given by^[^ ^58^ ^]^

(2)

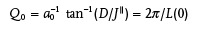

where *a*_0_ = 1.217 nm is the *c*‐axis lattice constant of CTS obtained through the X‐ray diffraction (XRD) spectra (Figure S2, Supporting Information). As the L‐TEM experiments show *L*(0)≈ 25 nm, we find *Q*_0_ = 0.025 Å^−1^ and *D*/*J* ^∥^ = 0.31. The values are considerably larger than those in CNS (*Q*_0_ = 0.013 Å^−1^, *D*/*J* ^∥^ = 0.16),^[^ ^58^ ^]^ indicative of the enhanced misalignment between interlayer spins in CTS. Furthermore, the analytical expression for the critical field at zero temperature is expressed as^[^ ^58^ ^]^

(3) $$\begin{array}{c} H_{c}(0)\;\;=\;\;{\left(\pi a_{0}Q_{0}/4\right)}^{2}J^{∥}S \end{array}$$

where *S* = 3/2 for the trivalent state of Cr atoms. As shown in Figure 2f, the critical field at low temperature (2 K) is about 14.1 kOe. Thus *H*_c_(0) can be estimated as 14.1 kOe, corresponding to 1.89 K (with the electron *g*‐factor *g* = 2). We then obtain *J* ^∥^ *S* ^2^ = 49 K and *DS* ^2^ = 15 K, both of which are larger than the values obtained from CNS (*J* ^∥^ *S* ^2^ = 18 K, *DS* ^2^ = 2.9 K).^[^ ^59^ ^]^ Therefore, we conclude that the enhancement of DM interaction is much greater than that of exchange interaction in CTS crystals, which should be responsible for the much shorter helical period in CTS compared to CNS.

### 2.4 Phase Diagram

**Figure 4:**
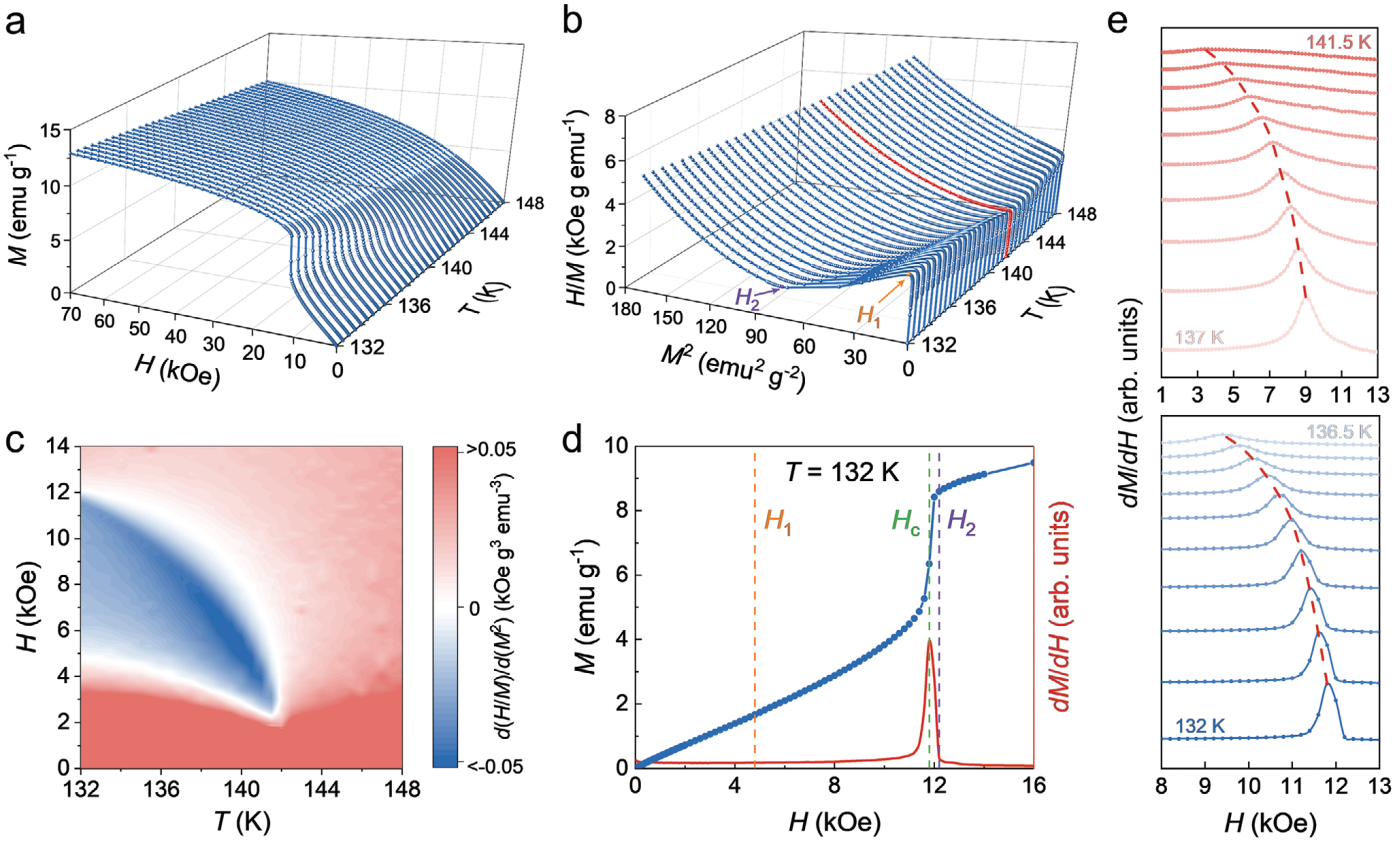
Magnetization of a CTS bulk single crystal around *T*_c_. a) Initial isothermal magnetization in the temperature region of 132–148 K. b) Corresponding *H*/*M* versus *M* ^2^ plots, wherein the curve of *T*_c_ = 142 K is highlighted in red. *H*_1_ and *H*_2_ are determined by the Banerjee criterion. c) Temperature‐ and field‐dependent d(*H*/*M*)/d(*M* ^2^) color map. d) *M*(*H*) curve (blue) and differential magnetization (red) measured at 132 K. *H*_c_ denotes the peak of the differential magnetization. e) Differential magnetization in the temperature region of 132–141.5 K with an increment of 0.5 K.

To shed light on the diversified magnetic behaviors in CTS, a comprehensive phase diagram as well as explicit phase boundaries is required. We utilized magnetization measurements, which have been proved as an effective technique for investigating such spin‐textured systems,^[^ ^29^, ^39^, ^60^, ^61^, ^62^, ^63^, ^64^, ^65^ ^]^ to determine the temperature‐ and field‐dependent phase diagram of CTS. **Figure** **4**a shows the initial isothermal magnetization of a high‐quality CTS bulk single crystal measured in the vicinity of *T*_c_ (132–148 K). At 132 K, an abrupt increase in the magnetic moment is observed before saturation, while it becomes less visible when the temperature increases and eventually fades away. The Banerjee criterion,^[^ ^66^ ^]^ which is based on the Landau mean‐field theory, is commonly used to identify the magnetic phase transition character around the transition point according to the signs of slopes of the *H*/*M* versus *M* ^2^ curves. Thereafter, the data presented in Figure 4a were replotted in the form of *H*/*M* versus *M* ^2^ (Figure 4b), wherein the curve measured at *T*_c_ = 142 K is highlighted in red. In the high‐field region, all the curves in Figure 4b have positive slopes, indicating a second‐order ferromagnetic to paramagnetic phase transition. However, below 142 K, there exists an intermediate region on each curve where the slope is negative, which is considered the CSL state that emerges between the initial CHM ground and final FFM states in the field‐increasing process. The two inflection points marked by *H*_1_ and *H*_2_ in Figure 4b refer to the critical fields of entering and exiting the CSL state, respectively. These two fields overlap each other immediately before 142 K, indicating that the *T*_c_ obtained from the *M*(*T*) peaks and the Banerjee criterion is self‐consistent and intrinsic. To observe the phase boundaries more clearly, we calculated the first derivative of the *H*/*M* versus *M* ^2^ curves and plotted them in Figure 4c. In this color map, the horn‐like blue zone, which is composed of negative values of d(*H*/*M*)/d(*M* ^2^), represents the CSL phase region and shrinks gradually with increasing temperature and disappears at *T*_c_ = 142 K.

**Figure 5:**
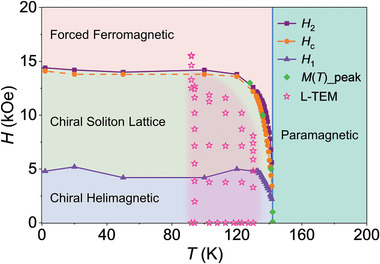
Magnetic phase diagram of CTS as a function of the magnetic field and temperature. *H*_1_ and *H*_2_ are determined by the Banerjee criterion, *H*_c_ is the peak position of the differential magnetization curve, the green squares are determined from the peak of the *M*(*T*) curves, and the pink stars denote the L‐TEM observations of chiral spin textures. The lowest temperature that can be reached in the L‐TEM experiments is 92 K.

Now, we discuss the aforementioned critical field *H*_c_ which is defined by the peak of the differential magnetization. Figure 4e shows the evolution of the d*M*/d*H* versus *H* curves with varying temperatures, and it is evident that *H*_c_ moves toward a lower magnetic field as the temperature increases. For clarity, we take 132 K as an example and annotate *H*_1_, *H*_c_, and *H*_2_ all together in Figure 4d. In the CHM ground state region, *H* < *H*_1_, the magnetization increases linearly with increasing field. The deviation from the linear dependence of *M* versus *H* in the intermediate zone, *H*_1_ < *H* < *H*_2_, can be explained by the fact that the field‐induced Zeeman energy competes with the DM interaction, leading to the breakdown of the perfect periodic spin spiral. Consequently, the ferromagnetic domains generate and separate the magnetic twists, which push the system into an incommensurate state, that is, the so‐called CSL state (Figure 1c). The ferromagnetic domains expand continuously with the increasing field and squeeze the solitons. As the applied magnetic field approaches *H*_c_, the magnetization increases abruptly because the spatial period of the CSL increases rapidly at this instant and most of the soliton twists degenerate. Thus, *H*_c_ can be regarded as a critical field wherein the system is situated in an extremely incommensurate condition. Furthermore, the nonlinear evolution ends at *H*_2_, which is approximately equal to the saturation field, above which, the CTS becomes a fully commensurate ferromagnetic system (Figure 1d). In this manner, we successfully established a comprehensive phase diagram for CTS based on magnetization measurements of bulk single‐crystal samples. In the phase diagram (**Figure** **5**), the spin textures observed in the L‐TEM experiments are appended by stars, and they nearly overlap with the CHM and CSL regions determined by the magnetization data, confirming the self‐consistency and reliability of our analyses.

In contrast to B20 magnets that have multiple DM vectors, CTS possesses a strong anisotropy derived from its hexagonal crystal structure, making it unlikely to form skyrmion textures.^[^ ^48^, ^49^ ^]^ Compared to the previously reported soliton magnet CNS, CTS has a much shorter helical period and a wider soliton phase region, and the CSL state can survive in an external magnetic field of up to ≈14 kOe. These features can be attributed to the stronger SOC of TaS_2_ than that of NbS_2_. It is also suggested that upon magnetic atom intercalation, the rich TMDC family constitutes an ideal platform to seek unconventional spin textures.

## 3 Conclusion

In summary, we successfully synthesized CTS, a magnetic‐atom‐intercalated TMDC having a chiral crystal structure. The coupling of the crystal chirality and strong SOC results in asymmetric DM interactions. As expected, we observed a helical spin texture with a short spatial period of ≈25 nm in CTS. When an external magnetic field is applied perpendicular to the helical axis, the CHM structure continuously transforms into the CSL state and finally the FFM state. Further, its evolution is consistent with the chiral sine‐Gordon theory, which opens promising perspectives for the application of CSL to fast‐speed nonvolatile magnetic memories. Furthermore, we established a comprehensive phase diagram based on analyses of the bulk magnetization data and L‐TEM observations. Benefiting from the layered crystal structure, CTS can be exfoliated down to 2D scale. Hence, CTS is expected to bring peculiar ingredients to the emerging fields of 2D spintronics and soliton physics.

## 4 Experimental Section

### Crystal Growth and Characterization

High‐quality CTS single crystals were grown using the chemical vapor transport method, similar to the authors’ recent report.^[^ ^13^ ^]^ Elemental Cr, Ta, and S powders were mixed at a molar ratio of 1.35:3:6 and sealed in a quartz ampoule using I_2_ as a transport agent. Thereafter, the quartz ampoule was evacuated and kept in a two‐temperature‐zone tube furnace, wherein the temperatures of the powder source and growth area were maintained at 1100 and 1000 °C, respectively, for 7 days. XRD spectra were collected on a Bruker D2 PHASER diffractometer using Cu *K_α_* radiation (λ = 1.5418 Å). The EDS pattern was acquired using a Zeiss MERLIN scanning electron microscope. The HRTEM image and electron diffraction pattern were obtained using an FEI Cs Image transmission electron microscope.

### Magnetization and Electrical Transport Measurements

Magnetization measurements were performed using an MPMS3 magnetometer (Quantum Design). Resistance measurements were carried out with a four‐terminal configuration. The electrodes on the bulk sample were made of gold wire and silver epoxy. The nanosheets were mechanically exfoliated onto a SiO_2_ (300 nm)/Si substrate using Scotch tape, and the thickness was verified using an atomic force microscope (Dimension Icon). Subsequently, the electrodes were patterned via electron‐beam lithography (Crestec‐9000), followed by e‐beam evaporation of Ti (10 nm)/Au (70 nm) metals. All the electrical measurements were performed in a Physical Property Measurement System (Quantum Design).

### Lorentz Transmission Electron Microscopy Imaging

The CTS lamellar samples were cut from bulk crystals and thinned by the FIB milling technique on an FEI Helios G4 UX scanning electron microscope and TESCAN LYRA3 FIB‐SEM system. The ion beam was shot into the bulk surface along the *c* direction and cut the sample along the *a* or *b* axis; thus the obtained lamella surface was *ac* or *bc* plane. However, no difference was observed between the *ac*‐ and *bc*‐plane specimens in the L‐TEM experiment. The thickness of the lamellae was ≈150 nm. The spin textures were observed using FEI Titan Cs Image and FEI Tecnai F30 transmission electron microscopes in the Lorentz–Fresnel mode with an acceleration voltage of 300 kV. The electron beam was perpendicular to the sample surface, and the typical defocus value was about −3 µm. Further, low‐temperature measurements were performed using a sample holder cooled by liquid nitrogen (Model 698, Gatan, USA). The perpendicular magnetic field was induced by gradually increasing the objective lens strength in small increments.

### Micromagnetic Simulations

The Mumax3 software package^[^ ^67^ ^]^ was used to perform the micromagnetic simulations and the unit cell size was set to 2 × 2 × 1 nm^3^. The material parameters were as follows: saturation magnetization *M*_s_ = 8.0 × 10^4^ A m^−1^, exchange stiffness constant *A* = 5.0 × 10^−12^ J m^−1^ and uniaxial magnetocrystalline anisotropy *K*_u_ = 5 × 10^3^ J m^−3^. To simulate the CSL with an asymmetric DM interaction, only the DM interaction in the *x* direction (helical axis) *D_x_* was considered and was set to ±3.0 mJ m^−2^ for the right/left‐handed CSL. The corresponding L‐TEM contrast patternswere simulated using the MALTS code via the Aharonov–Bohm expression.^[^ ^68^, ^69^ ^]^

## Conflict of Interest

The authors declare no conflict of interest.

## Supporting information

Supporting Information

## Data Availability

The data that support the results of this study are available from the corresponding author upon reasonable request.
