# Supplementary material for: Chiral Helimagnetism and One‐Dimensional Magnetic Solitons in a Cr‐Intercalated Transition Metal Dichalcogenide
Source: Adv Mater. 2021 Jul 24;33(35):2101131. doi: 10.1002/adma.202101131 (PMC11468724; doi:10.1002/adma.202101131)
Supplement: Supplementary file 1 — Supporting Information [file ADMA-33-2101131-s001.pdf]

© 2021 Wiley-VCH GmbH

ADVANCED MATERIALS

Supporting Information

for *Adv. Mater.,* DOI: 10.1002/adma.202101131

Chiral Helimagnetism and One-Dimensional Magnetic Solitons in a Cr-Intercalated Transition Metal Dichalcogenide

Chenhui Zhang, Junwei Zhang, Chen Liu, Senfu Zhang, Ye Yuan, Peng Li, Yan Wen, Ze Jiang, Bojian Zhou, Yongjiu Lei, Dongxing Zheng, Chengkun Song, Zhipeng Hou, Wenbo Mi, Udo Schwingenschlögl, Aurélien Manchon, Zi Qiang Qiu, Husam N. Alshareef, Yong Peng, and Xi-Xiang Zhang**

Supporting Information

Chiral helimagnetism and one-dimensional magnetic solitons in a Cr-intercalated transition metal dichalcogenide

Chenhui Zhang, Junwei Zhang, Chen Liu, Senfu Zhang, Ye Yuan, Peng Li, Yan Wen, Ze Jiang, Bojian Zhou, Yongjiu Lei, Dongxing Zheng, Chengkun Song, Zhipeng Hou, Wenbo Mi, Udo Schwingenschlögl, Aurélien Manchon, Zi Qiang Qiu, Husam N. Alshareef, Yong Peng and Xi-Xiang Zhang**

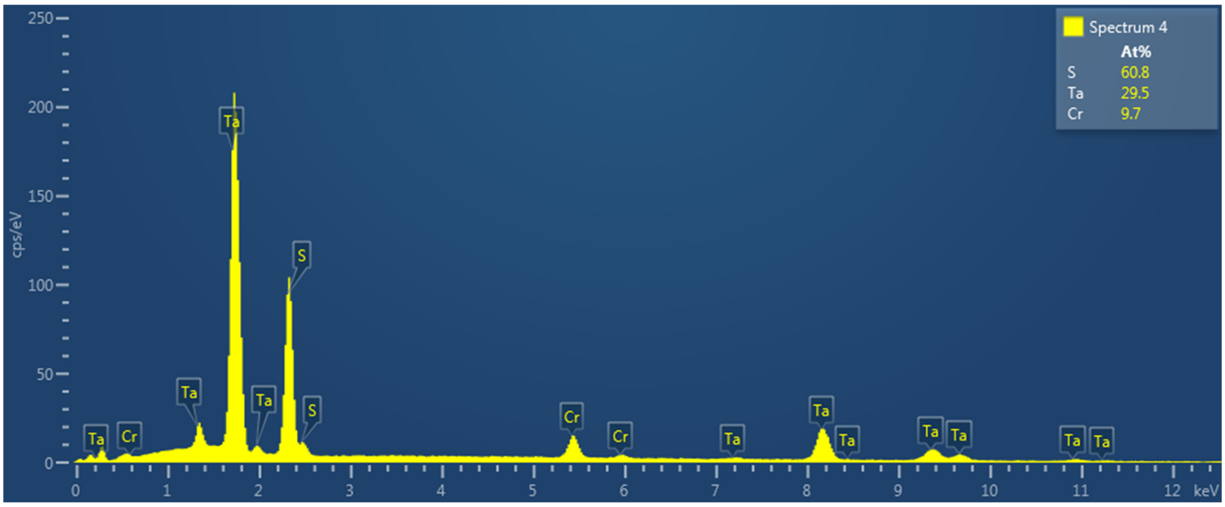

Figure S1. Energy-dispersive X-ray spectrum of an as-grown single-crystal sample. An atomic percentage ratio of Cr:Ta:S = 9.7:29.5:60.8 was acquired, demonstrating a chemical composition of $Cr_{0.33}TaS_{2.06}$.

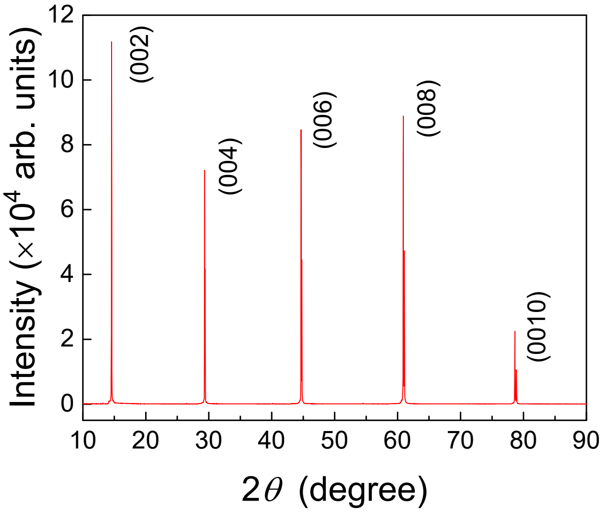

Figure S2. Single crystal X-ray diffraction pattern of a CTS bulk sample. The spectra were collected at room temperature with the crystal flake lying flat on the sample holder, in which the peaks are indexed as (00*L*) planes.

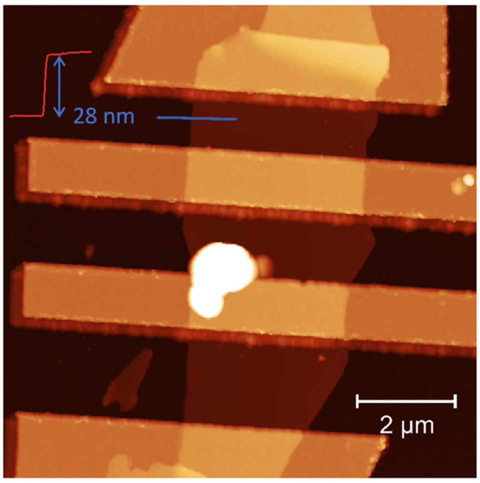

Figure S3. AFM image of a CTS nanosheet-based device.

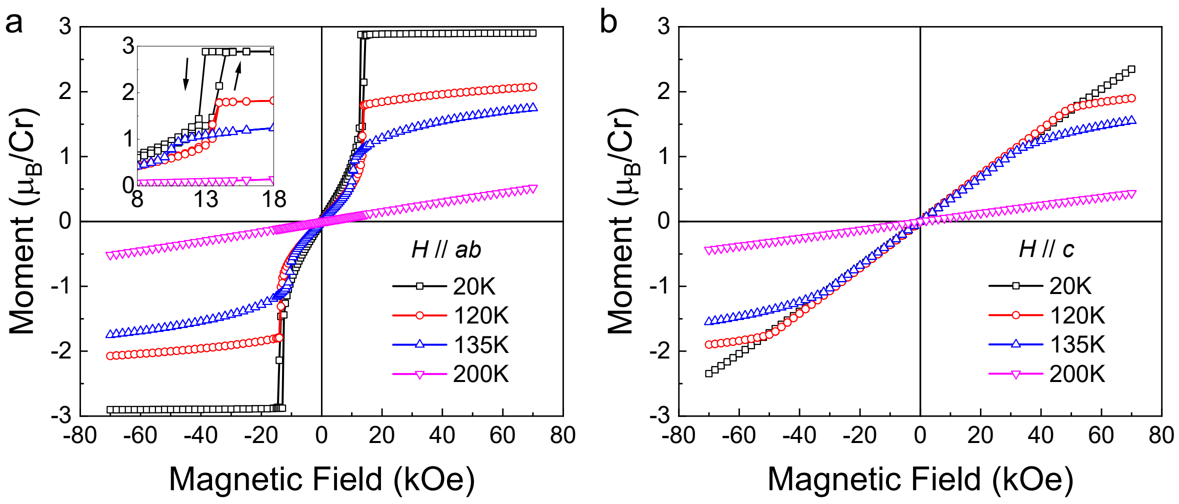

Figure S4. Magnetization of a CTS bulk single crystal by sweeping the field between −70 kOe and 70 kOe at various temperatures. a) The magnetic field is applied in the *ab* plane. Since there is a neglegiblly weak anisotropy in the *ab* plane, the $M(H)$ curves with any angle between H and a axis is nearly the same. The field range of 8–18 kOe is magnified and shown in the inset, where the arrows indicate the magnetic field sweeping directions. b) The magnetic field is applied along the c axis.

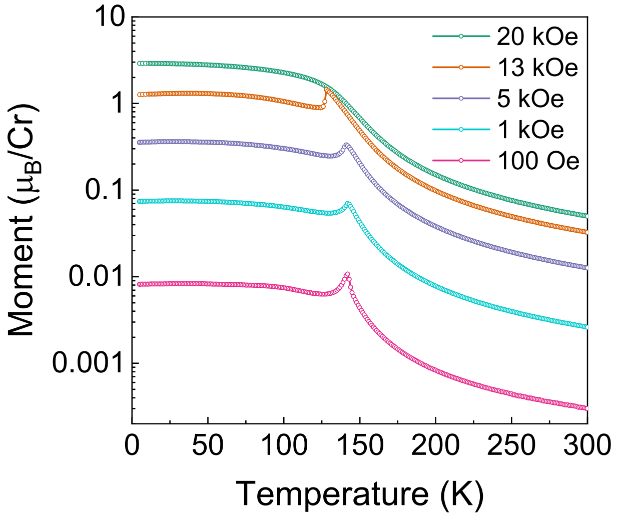

Figure S5. Log-scale plot of temperature-dependent magnetization of a CTS bulk single crystal measured with zero-field-cooled protocol at various magnetic fields applied in the *ab* plane.

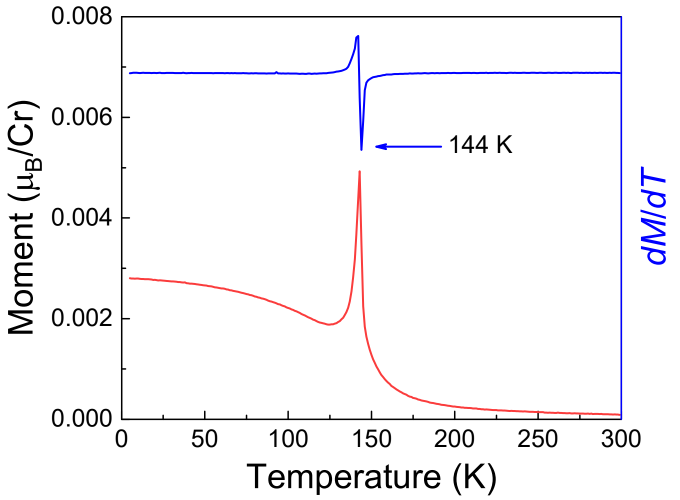

Figure S6. Temperature-dependent magnetization as well as *dM*/*dT* curve of a CTS bulk single crystal measured with 50 Oe field-cooled protocol in the *ab* plane.

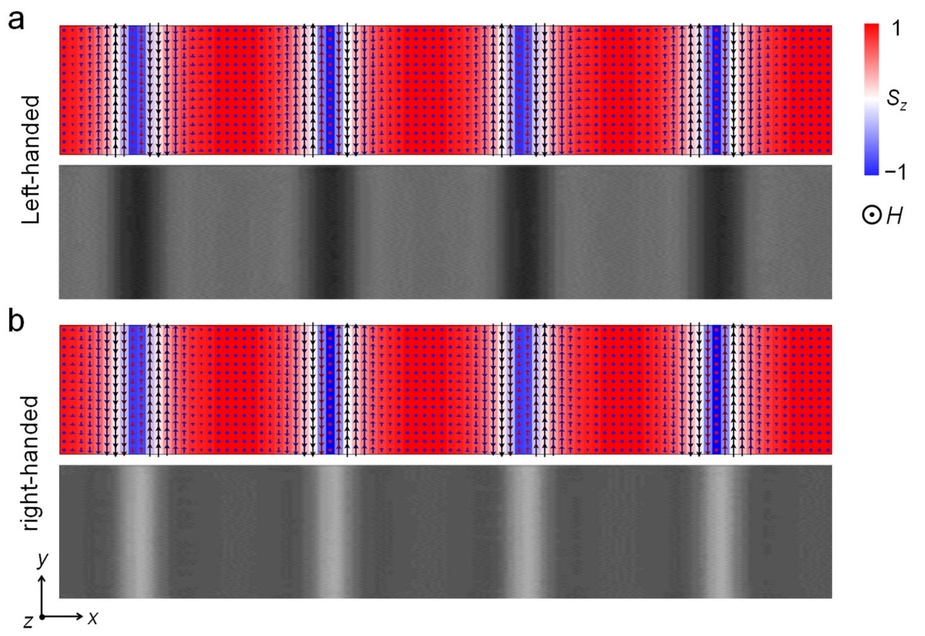

Figure S7. | Illustrations of CSLs as well as the corresponding simulated L-TEM patterns in Fresnel under-focused mode. a) The pattern of the left-handed CSL shows an alternating wide grey and narrow dark contrast. b) The pattern of the right-handed CSL shows an alternating wide grey and narrow bright contrast. The external magnetic field is applied along the *z* axis, and the *x* axis is the helical axis.

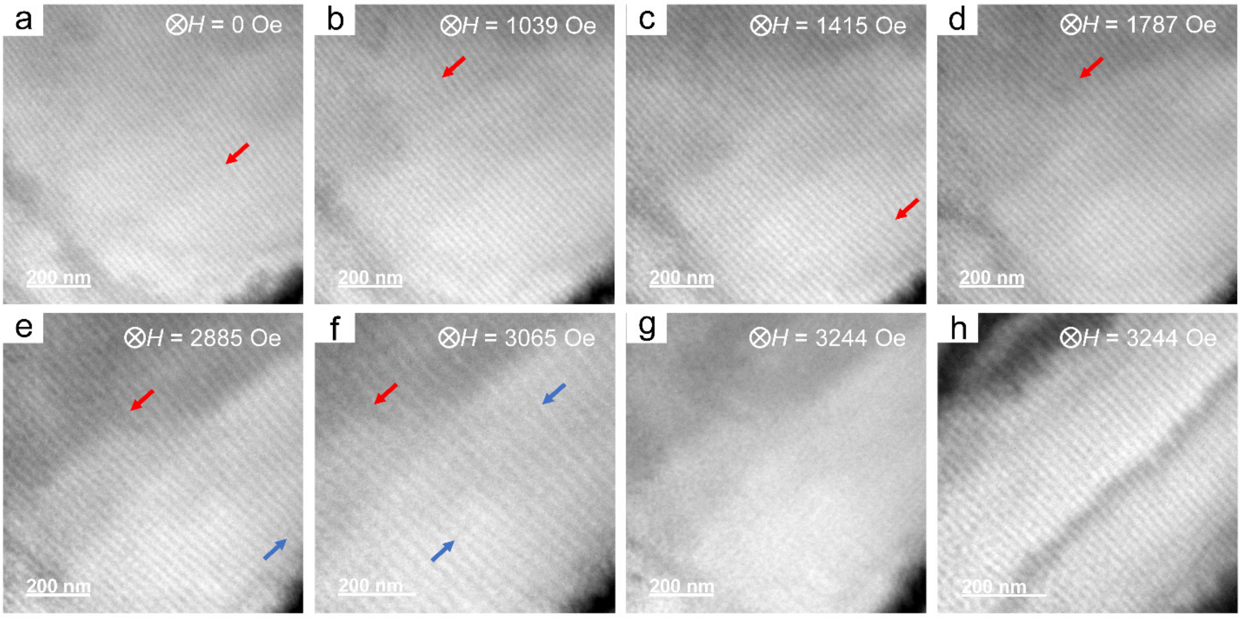

Figure S8. Magnetic lattice dislocations and electron illumination effect on the CSL–FFM transition. a-g) The magnetic-field-dependent evolution of spin textures at 94 K. The viewing area is kept illuminated during the whole process. h) A micrograph taken from another area of the sample immediately after (g) with the temperature and field conditions kept unchanged. The red arrows indicate the motion of a magnetic lattice dislocation. The blue arrows indicate the dislocations arising during the field-increasing process.

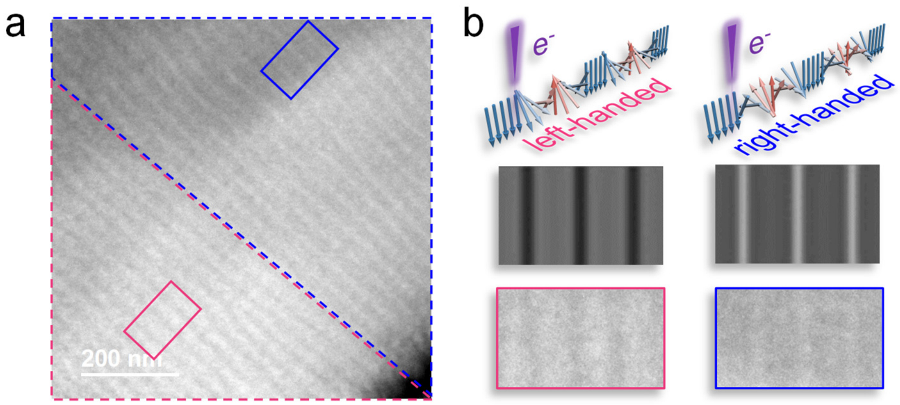

Figure S9. The reversal of chirality at a grain boundary. a) A L-TEM image reproduced from Figure S8f. The pink and blue dashed lines enclosed areas are separated by a grain boundary where the spin chiralities of solitons are reversed. b) Schematic diagrams of CSL with left- and right-handed chiralities. The middle panel is the expected contrast patterns in Fresnel under-focused mode. The observed patterns in the lower panel are captured from corresponding areas in (a).

Supplementary Note 1. Electron illumination effect on the CSL–FFM transition

We found that electron illumination has a significant impact on the CSL–FFM transition field in L-TEM experiments. To avoid this extrinsic effect as much as possible, in the interval of acquiring the micrographs in Figure 3c, the sample is moved away from the electron beam, by which the CSL–FFM transition was observed at ~12.7 kOe. On the contrary, at the same temperature, if the viewing area of the sample is kept illuminated during the whole process, the transition field will be significantly reduced to ~3.2 kOe, as shown in Figure S8g. In the meantime, the stripe patterns still remain in the other areas of the sample that are not illuminated by the electron beam (see Figure S8h).

Supplementary Note 2. Magnetic lattice dislocations and grain boundaries with reversed chiralities

We observed magnetic lattice dislocations in some areas of the L-TEM samples, which are indicated by the arrows in Figure S8. This special structure also appears in CNS and it mediates the formation of CSL and FFM regions[1]. As the magnetic field is increased gradually, more dislocations arise (blue arrows). The motion of dislocations pointed by the red arrows is recorded, and it seems unidirectional. More strikingly, we find that the motion path is along a grain boundary where the magnetic chirality of soliton is reversed, as presented in Figure S9. The magnetic pattern in the area enclosed by the pink dashed lines is composed of wide grey and narrow dark stripes, while the opposite situation (namely, wide grey and narrow bright) is observed in the area enclosed by the blue dashed lines. These two different configurations of contrast can be deciphered by two kinds of soliton lattices with left-handed and right-handed chiralities[2], which have been explained in Figure S7. We speculate that racemic twinned grains were formed in CTS during the crystal growth, which is commonly seen in chiral crystals[2-5].

Supplementary References

[1] G. W. Paterson, T. Koyama, M. Shinozaki, Y. Masaki, F. J. T. Goncalves, Y. Shimamoto, T. Sogo, M. Nord, Y. Kousaka, Y. Kato, S. McVitie, Y. Togawa, *Phys. Rev. B* **2019**, 99, 224429.

[2] Y. Togawa, T. Koyama, Y. Nishimori, Y. Matsumoto, S. McVitie, D. McGrouther, R. L. Stamps, Y. Kousaka, J. Akimitsu, S. Nishihara, K. Inoue, I. G. Bostrem, V. E. Sinitsyn, A. S. Ovchinnikov, J. Kishine, *Phys. Rev. B* **2015**, 92, 220412(R).

[3] K. Adachi, N. Achiwa, M. Mekata, *J. Phys. Soc. Jpn.* **1980**, 49, 545.

[4] Y. Horibe, J. Yang, Y. H. Cho, X. Luo, S. B. Kim, Y. S. Oh, F. T. Huang, T. Asada, M. Tanimura, D. Jeong, S. W. Cheong, *J. Am. Chem. Soc.* **2014**, 136, 8368.

[5] A. Inui, R. Aoki, Y. Nishiue, K. Shiota, Y. Kousaka, H. Shishido, D. Hirobe, M. Suda, J. I. Ohe, J. I. Kishine, H. M. Yamamoto, Y. Togawa, *Phys. Rev. Lett.* **2020**, 124, 166602.
